# Application of ultrasound in the closed reduction and percutaneous pinning in supracondylar humeral fractures

**DOI:** 10.1186/s13018-022-02982-0

**Published:** 2022-02-11

**Authors:** Andreas Rehm, Joshua C. Y. Ong, Tamás Kobezda, Elizabeth Ashby

**Affiliations:** Department of Paediatric Orthopaedics, Paediatric Division, Cambridge University Hospitals NHS Trust, Hills Road, Cambridge, CB2 0QQ UK

**Keywords:** Supracondylar humerus fracture, Supracondylar fracture, Supracondylar humeral fracture, Gartland classification, Lateral pinning, Percutaneous pinning, Paediatric elbow trauma, Pediatric elbow trauma, Anterior humeral line, Lateral capitellohumeral angle, Baumann’s angle.

## Letter to the Editor

We read with interest the recent publication by Wu et al. [1]. Wu et al. [1] stated in their abstract that they treated 64 children with supracondylar humerus fractures with ultrasound-guided closed reduction and percutaneous pinning, which is then refuted in the method and discussion section where the authors stated that intraoperative radiographs were needed for placement of the pins because of the difficulties to delineate the pins with ultrasound (US). Wu et al. [1] presented 2 cases in their Figs. 1 and 2 where it is claimed that the presented US images of the distal humerus show that both fractures were reduced back to its normal position. However, the image of what was described as a “lateral” radiograph in the authors’ Fig. 1 shows obvious displacement of the medial column with malrotation, with it neither being possible to measure the capitellohumeral angle, nor to assess the relationship between the anterior humeral line and the capitellum because of it being an oblique radiograph. The lateral radiograph in the authors’ Fig. 2 shows an extension and rotational deformity of the distal fragment with the anterior humeral line (AHL) only touching the front of the capitellum. These two cases, where clearly visible malreductions were described as normal, raise doubts about the reliability of Wu et al.’s [1] radiographic assessments and indicate that ultrasound might not be feasible to judge the accuracy of the reduction.

Wu et al. [1] stated in their discussion that the anterior humeral line passes through the anterior third of the capitellum, which is contrary to the findings by Ryan et al. [2] who identified that the AHL ran through the middle third of the capitellum in 100% of children ≥ 5 years of age and that it fell outside the middle third in 25% of children < 5 years of age (anterior third: 15.3%; posterior third: 9.7%).

Wu et al. [1] did not state how many surgeons assessed the radiographs, did not perform intra and interobserver reliability testing for their measurements and did not assess correction of rotational deformities by measuring the lateral rotation percentage as described by Gordon et al. [3]. Shank et al. [4] reported a mean lateral capitellohumeral angle (LCHA) of 50.8° ± 6°, with an intraobserver correlation coefficient (CC) of 0.67 and interobserver CC of 0.37, but stated that a sagittal angulation abnormality of at least 12° (< 39° or > 63°) is necessary to be confident that the change is not because of measurement error alone. Hasegawa et al. [5] reported a mean LCHA of 45° (range 22°–70°), with intraobserver CC of 0.77 and interobserver CC of 0.535. The former indicates that the LCHA is possibly not a good measure to judge the quality of reduction because of the wide normal range and relatively low intra and interobserver reliabilities, with radiographs of the other elbow usually not being available for comparison.

Camp et al. [6] measured that the perceived Baumann’s angle (BA) increases with internal and decreases with external humeral rotation if an anteroposterior radiograph (APR) is not taken as a true APR, with the perceived angle changing by ~ ± 1.6° per 10° change of rotation with the humerus parallel to the collector/cassette and by ~ ± 5° per 10° change of rotation with the humerus flexed 30°. Therefore, rotational deformities will result in BA measurement inaccuracies, with internal malrotation of the distal fragment producing an apparent increase in BA.

Wu et al. [1] provided one off radiographic and clinical measurements at a mean follow up of 19.4 months without data on recovery of range of movement over time and comparison between intra-operative post-reduction and follow-up images, despite 6 follow-up visits for each patient, so that we do not know if the inadequate reductions under US guidance, as seen on the provided images, resulted in prolonged functional recovery compared to the non-US group.

In conclusion, Wu et al.’s provided radiographic evidence shows that the authors did not appreciate rotational deformities, with these being described as normal, which raises questions about the reliability of the provided radiographic measurements, with the use of ultrasound assistance possibly resulting in an increased rate of inadequate supracondylar humeral fracture reductions. This is in addition to the 42% mean increase of costs associated with the use of US because of the extra time under general anaesthetic in theatre, with Wu et al. [1] not having provided evidence that US resulted in a clinically relevant reduction of radiation.

## Data Availability

Not applicable.

## Abbreviations

AHL: Anterior humeral line
APR: Anteroposterior radiograph
BA: Baumann’s angle
CC: Correlation coefficient
LCHA: Lateral capitellohumeral angle
US: Ultrasound
